# 

**Published:** 1890-10

**Authors:** 


### NOTICE TO CONTRIBUTORS.

We are glad to receive contributions from every one who knows anything of interest to the profession. Articles designed for publication in the JOURNAL should be handed in before the first day of the month.

The Editors are not responsible for the views or opinions of contributors.

All communications should be addressed to

284 FRANKLIN STREET,

BUFFALO, N. Y.

